# Concentration of Cd, Pb, Hg, and Se in Different Parts of Human Breast Cancer Tissues

**DOI:** 10.1155/2014/413870

**Published:** 2014-02-11

**Authors:** Mehrnoosh Mohammadi, Alireza Riyahi Bakhtiari, Saber Khodabandeh

**Affiliations:** ^1^Environmental Forensic Laboratory, Department of Environmental Sciences, Faculty of Natural Resource and Marine Science, Tarbiat Modares University, P.O. Box 64414-356, Noor, Mazandaran, Iran; ^2^Faculty of Marine Science, Tarbiat Modares University, Tehran, Iran

## Abstract

Breast cancer is the major cause of cancer morbidity and mortality between women in the world. Metals involved in environmental toxicology are closely related to tumor growth and cancer. On the other hand, some metals such as selenium have anticarcinogenic properties. The aim of this study is to determine the concentration of cadmium, lead, mercury, and selenium in separated parts of tegmen, tumor, tumor adiposity, and tegmen adiposity of 14 breast cancer tissues which have been analyzed by graphite furnace atomic absorption (AA-670) and ICP-OES (ULTIMA 2CE). Our results show that Se and Hg have maximum and minimum concentration, respectively. Statistical analysis reveals no significant differences between metal accumulations in different parts of cancer tissues (*P* > 0.05) and this observation might be due to the close relation of separated parts of fatty breast organ. Thus, we could conclude that a high level of these heavy metals is accumulated in Iranian cancerous breasts and their presence can be one of the reasons of cancer appearance.

## 1. Introduction

Breast cancer is the most frequently diagnosed cancer and the leading cause of cancer death in women worldwide, accounting for 23% (1.38 million) of the total new cancer cases and 14% (458,400) of the total cancer deaths in 2008. About half of the breast cancer cases and 60% of the deaths are estimated to occur in economically developing countries [1]. In Iran, the incidence of breast cancer is rising and ranked first among malignancies in women. Also, patients with advanced stages of the disease are relatively younger (about 10 years) than their western counterparts [2, 3].

Exposure to environmental pollutants such as metals including cadmium, chromium, nickel, and arsenic is classified in Group 1 of the International Agency for Research on Cancer categories of carcinogen [4]; it also reports lead as a suspected human carcinogen (Group 2A) [5] and also mercury as possibly carcinogenic to humans (Group 2B) [6].

In order to explain the role of metals in breast cancer incidence, we should refer to the studies in the field of estrogenicity of metals that express estrogen-like activity in breast cancer cells and suggest several pathways to explain association of metals with human cancer [7–11]. On the other hand, cadmium, lead, and mercury as carcinogens belong to the group of selenium, the antagonistic elements that compete with selenium uptake as anticarcinogen [12]. The mechanism of Se as an anticarcinogenic element is unknown, but several speculative hypotheses have been advanced [13]. Se exerts its essential role in the formation of glutathione peroxidase, a selenoenzyme that protects body against oxidative injury and free radical damage so its suggested mechanism for cancer prevention includes effects upon programmed cell death, DNA repair, carcinogen metabolism, and the immune system [14–17]. Therefore, it seems that, according to the results of these researches, a considerable amount of literature has been published on the determination of metals in human breast cancer tissues which show various values in malignant and benign tissues in comparison with healthy tissues [17–28]. In addition, all available studies have only focused on metal concentration totally in breast tissue, but the aim of this study is to determine Cd, Pb, Hg, and Se in various parts of breast tissue (tegmen, tumor tissue, tumor adiposity, and tegmen adiposity) in order to compare any significant differences which may exist between different parts of breast tissues. Also, this research may be the first report of metal concentration in Iranian breast cancer tissues.

## 2. Material and Methods

In order to determine Cd, Pb, Hg, and Se concentrations in different parts of malignant breast tissue, 14 removed samples by mastectomy surgery of women patients (in age range 30–50) were taken from Imam hospitals located in Uremia and separate tegmen, tumor, tumor adiposity, and tegmen adiposity of breast cancerous tissues were dried in freeze-dryer at −64°C for 20 to 30 hours. Then, about 1 g of each part of separated breast tissue was put in the digestion tubes (polytetrafluoro Ethylene) with 5 mL concentrated HNO_3_ for 3 hours in 100°C on hot block digester until the disappearance of brown fumes. After cooling, 1 to 3 mL H_2_O_2_ 30% for 1 hour on heater was added. After cooling, it was filtered with Whatman filter paper number 1 and then diluted with deionized water to final volume of 25 mL [26, 30].

Pb and Cd measurements were performed by using AAS and with the graphite furnace technique model AA-670 and Se analysis was done by ICP-OES (ULTIMA 2CE). Metal concentrations in breast tissues were calculated as *μ*g/kg of dry weight.

The AAS instrument was calibrated using aqueous standards of 10, 30, and 60 *μ*g/kg for Pb and 0.5, 2, and 5 *μ*g/kg for Cd in breast cancer tissues. There was a good linear relation between absorbance and standard concentrations of Pb and Cd. Linearity was evaluated by calculating the *R*-square value, which was 0.998 for Pb and 0.999 for Cd.

The detection limit with AAS was calculated as 3 times the SD of the blank sample divided by the slope of calibration curves (1.57 *μ*g/kg for Pb and 0.18 *μ*g/kg for Cd). The detection limit of Se with ICP-OES was 0.2 *μ*g/kg. About 0.02–0.04 g of dried samples was put on nickel boot for measuring Hg by Leco AMA 254 Advanced Mercury Analyzer (USA).

To evaluate the analytical potency of the proposed methodology, accuracy of total Hg analysis was checked by running three samples of Standard Reference Materials (SRM), National Institute of Standards and Technology (NIST), SRM 1633b, SRM 2709, and SRM 2711 in seven replications. Recovery was between 95.3% and 101%. The detection limit of the used instrument was 1 *μ*g/kg of dry weight. To check for contamination, all of used glassware was acid-washed and one blank was analyzed after five samples. The accuracy of method was tested by analyzing (SRM, OL-96). The analytical values were in the range of certificated values. Recoveries were consistently in the range 91–98%.

## 3. Results and Discussion

Table 1 presents mean, standard deviation, and range of Pb, Cd, Hg, and Se concentration in 4 parts of tegmen, tumor, tumor adiposity, and tegmen adiposity of 14 breast cancer tissues. As it can be seen in the table, the maximum and minimum obtained values were Se and Hg, respectively. The statistical analysis of data has been done by SPSS version 17 software. The concentration data of Pb in Kolmogorov-Smirnov test was not normal (*P* < 0.05), so by using the Kruskal-Wallis nonparametric test, no significant differences between Pb values in parts of the breast tissues were shown (*P* = 0.820). To assay the differences between Cd, Se, and Hg, one-way ANOVA test was used and no significant differences were found between metals in the separated breast cancer tissues (resp., *P* = 0.322, *P* = 0.235, and *P* = 0.148).

No significant difference between the concentrations of metals in four separate parts might be due to the very close blood relationship between tissues. A noticeable point in this research is that the preparation of healthy tissues as control samples for comparing cancer tissues was not possible, so we compared our results with previous studies. Despite the possible relation between Cd and breast cancer exposure, its values in different parts of 14 breast cancer samples show mean concentration in tegmen (35.51 *μ*g/kg), tumor (45.04 *μ*g/kg), tumor adiposity (41.15 *μ*g/kg), and tegmen adiposity (32.95 *μ*g/kg).

Table 2 compares Cd, Pb, and Se concentration in breast tissues from results of available studies. Antila et al. [20] surveyed fatty breast and healthy tissues, so there were not any significant differences between Cd in cancerous (20470 *μ*g/kg) and healthy (31700 *μ*g/kg) ones and the maximum concentration of Cd among hitherto accessible studies has been reported. The separation of the close parts of breast tissues in this study did not show any significant difference between them and, as it can be inferred from the table, the present results are in accordance with the results of Strumylaite et al. [27] that report the minimum Cd values so far.

Due to the multiple carcinogenic evidence of lead, its detection has been done in various tumors such as breast ones. According to Table 2, the considerable concentrations in tegmen (336.18 *μ*g/kg), tumor (327.50 *μ*g/kg), tegmen adiposity (396.52 *μ*g/kg), and tumor adiposity (365.73 *μ*g/kg) are similar to the results of Majewska et al. [21] and Kubala-kukuś et al. [23]. Alatise and Schrauzer [17] reveal the minimum concentration which has been reported so far.

Mercury has been less considerable as a carcinogenic metal. Estrogenic responses of low concentration of methylmercury stimulate breast cells growth. Also mercuric chloride has been widely considered as causative of tumors [9]. Among collected studies, Rizk and Sky-Peck [18] report the mean concentration of Hg (770 *μ*g/kg dry weight) in breast cancer tissues and, according to the results of this study, it is supposed that the accumulation of this metal in tegmen (29.10 *μ*g/kg), tumor (33.26 *μ*g/kg), tumor adiposity (28.04 *μ*g/kg), and tegmen adiposity (26.13 *μ*g/kg) can have a role in carcinogenicity.

As it can be seen in Table 2, according to the studies, the accumulation of Se in breast cancer tissues has been determined. The mean concentration of Se is similar to the results of Rizk and Sky-Peck [18], Kue et al. (2002), and Alatise and Schrauzer [17].

The noticeable issue is that metals are just one of the effective factors in carcinogenesis or anticarcinogenesis, so their clear mechanism would have been investigated. This study just reports the concentration of some metals in breast cancer in women's samples from selective hospital, as we know that different factors may affect the occurrence of cancer especially breast cancer in women all over the world and environmental pollutants such as metals from several sources could enter the human body and, by accumulation, increase, and intensification, they may cause the incidence of cancer.

## 4. Conclusion

This study showed that there were not any significant differences between metals concentration in different parts of breast cancer tissues. This result might be because of close relation of separated parts of fatty breast organ. In general, in puberty and presence of estrogen hormone, breast cells have been grown rapidly. In normal situation, after sudden increased rate of estrogen and breast cell growth, hormone balance became in equilibrium and irregular cell proliferation was interrupted. According to recent researches and hypotheses, it could be concluded that estrogen-like properties of metals could mostly influence hormonal responses by binding to estrogen receptors and disrupt endocrine system and finally increased proliferation of cells would be occurring. We conclude, thus, that a high level of these heavy metals is accumulated in Iranian cancerous breasts and their presence can be one of the reasons for breast cancer appearance.

## Figures and Tables

**Table 1:** Mean, standard deviation, and range of metal concentration (*μ*g/kg) in 4 parts of tegmen, tumor, tumor adiposity, and tegmen adiposity of 14 breast cancer tissues.

Metals	Cancer tissues	Mean	Standard deviation	Range
Cadmium	Tegmen	35.51	14.43	22.14–77.36
	Tumor	45.04	18.85	25.14–87.93
	Tumor adiposity	41.15	18.42	21.83–77.77
	Tegmen adiposity	32.95	6.27	21.25–38.54

Lead	Tegmen	336.18	119.00	215.72–609.84
	Tumor	327.5	96.37	246.47–536.71
	Tumor adiposity	396.52	176.94	232.68–839.27
	Tegmen adiposity	365.73	138.35	216.63–617.61

Mercury	Tegmen	29.10	10.30	16.90–52.52
	Tumor	33.26	16.08	19.04–79.42
	Tumor adipose	28.04	7.13	17.85–44.24
	Tegmen adipose	26.13	7.01	15.86–39.12

Selenium	Tegmen	767.60	336.36	56.22–1219.18
	Tumor	1040.26	562.12	479.97–2226.86
	Tumor adipose	823.88	320.54	248.57–1367.14
	Tegmen adipose	731.79	397.35	41.07–1426.38

**Table 2:** A comparison between mean Cd, Pb, and Se concentration (*μ*g/kg) in breast cancer tissues in our research with previous studies.

Reference	Number of Samples	Conc. of Cd	Conc. of Pb	Conc. of Se
Rizk and Sky-Peck, 1984 [18]	26		1.55 ± 1.24 (D.W)	1.02 ± 0.43 (D.W)
Tariq et al., 1995 [19]	2	0.36 (W.W)	1.36 (W.W)	
Antila et al., 1996 [20]	43	20.4 ± 17.5 (D.W)		
Majewska et al., 1997 [21]	26		0.331 ± 0.083 (D.W)	0.121 ± 0.022 (D.W)
Kuo et al., 2002 [29]	68			1.05 ± 0.47 (D.W)
Siddiqui et al., 2006 [22]	25		0.54 ± 0.12 (D.W)	
Majewska et al., 2007 [25]	26			0.156 ± 0.075 (D.W)
Kubala-kuku$\overset{´}{\text{s}}$ et al., 2007 [23]	26		0.335 ± 0.289 (D.W)	
Pasha et al., 2008 [26]	53	1.21 ± 2.64 (W.W)	8.32 ± 13.2 (W.W)	
Alatise and Schrauzer, 2010 [17]	12		0.11 (D.W)	0.96 (D.W)
Strumylaite et al., 2011 [27]	51	0.053 (D.W)		
Romanowicz-Makowska et al., 2011 [28]	67	0.76 ± 0.38 (D.W)		
Our research				
Tegmen	14	35.51 ± 14.43 (D.W)	336.18 ± 119 (D.W)	767.6 ± 336.36 (D.W)
Tumor	14	45.04 ± 18.85 (D.W)	327.50 ± 96.37 (D.W)	1040.26 ± 562.12 (D.W)
Tumor adiposity	14	41.15 ± 18.42 (D.W)	396.52 ± 176.94 (D.W)	823.88 ± 320.54 (D.W)
Tegmen adiposity	14	32.95 ± 6.27 (D.W)	365.73 ± 138.35 (D.W)	731.79 ± 397.35 (D.W)

D.W: dry weight; W.W: wet weight.
